# Should the Government Be Allowed to Take Control over Your Car as Part of a Disaster Management Plan?

**DOI:** 10.3390/ijerph17217780

**Published:** 2020-10-24

**Authors:** Yipeng Lv, Zafar Zafari, Boshen Jiao, Casey Chun, Lulu Zhang, Zhaoxin Wang, Peter Alexander Muennig

**Affiliations:** 1 The Department of Social Medicine and Health Service Management, School of Medicine, Shanghai Jiaotong University, Shanghai 200025, China; epengl@sjtu.edu.cn; 2 Global Research Analytics for Population Health (GRAPH), Mailman School of Public Health, Columbia University, New York, NY 10027, USA; zzafari@rx.umaryland.edu (Z.Z.); jiaoboshen@gmail.com (B.J.); cc4196@cumc.columbia.edu (C.C.); 3 Department of Pharmaceutical Health Services Research, University of Maryland School of Pharmacy, Baltimore, MD 21250, USA; 4 The Comparative Health Outcomes, Policy, and Economics (CHOICE), School of Pharmacy, University of Washington, Seattle, WA 98195, USA; 5 The Department of Military Health Service Management, College of Military Health Service Management, Second Military Medical University, Shanghai 200433, China; zllrmit@163.com

**Keywords:** autonomous vehicles, disaster management, earthquake emergency rescue, risk reduction

## Abstract

Introduction: With the Safety Ensuring Lives Future Deployment and Research in Vehicle Evolution (SELF DRIVE) Act in the United States, there is a growing interest in autonomous vehicles (AVs). One avenue of innovation would be to use them to mobilize and coordinate response efforts during natural disasters. This study uses an earthquake response in an urban, developed setting as a hypothetical example case study. In this hypothetical scenario, private AVs would be mobilized to help rescue victims from collapsed structures. Methods: A Markov model compared an intervention arm with AVs to a status quo arm using a hypothetical cohort of American earthquake victims. The three possible health states were trapped but alive, rescued and alive, and dead. The cycle length of the Markov model was 6 h. Results: The cost of deploying AVs was $90,139 relative to $87,869 in status quo arm. Using AVs produced an incremental cost of $2269 (95% credible interval (CI) = $−12,985–$8959). Victims have 7.33 quality-adjusted life years (QALYs) in the intervention arm compared to 7.20 QALYs in the status quo arm, resulting in an incremental gain of 0.13 (95% CI = −0.73–2.19) QALYs. The incremental cost-effectiveness ratio (ICER) was $16,960/QALY gained (95% CI = cost-saving–$69,065/QALY). Discussion: The mobilization of private AVs in the setting of an earthquake has the potential to save money and reduce the loss of life. AVs may advance emergency management competencies.

**Highlights**

1. This article forwards a thought experiment in which the trade-off between the costs and benefits of using privately owned autonomous vehicles (AVs) in disaster management situations are assessed.
2. Using a simulation model, we assess the cost-effectiveness of deploying private AVs immediately following a major earthquake as a case study.
3. Our case study is limited by the large number of assumptions required surrounding a technology that is still maturing.
4. Our study suggests that the government needs to begin developing policies that extend beyond the legal and safety impacts of AV use.

## 1. Introduction

Autonomous vehicles (AVs) were put on the roads of global cities in 2018, and are sometimes tested without a human behind the wheel [1,2,3]. The US, Germany, the UK, the Netherlands, South Korea, Australia, and China are examples of nations that have allowed AVs to mix with human piloted vehicles (HPVs) on public streets. However, legislation governing the use of AVs tends to be focused on their safety. The broader consequences of (and possibilities for) widespread AV use also need to be considered.

In the United States, the Safely Ensuring Lives Future Deployment and Research in Vehicle Evolution (SELF DRIVE) Act (HR 3388) is designed to standardize and establish a federal role in regulating the safety of AVs. This law is entirely focused on collisions, licensing, and insurance [1]. A similar approach was taken when “horseless carriages” were introduced on the road in the 1900s. Had legislators then foreseen the rise of pollution and congestion when formulating automobile policy, many lives might have been saved by better planning the transition from railways to roadways. Likewise, AV legislation should consider the broader range of impact AVs will have on society as a whole, both good and bad. We provide a case study of one potential use for privately owned AVs in disaster management situations.

Specifically, we explore the costs and benefits associated with legislation that allows the government to take control of private AVs following an earthquake. We posit that integrating AV software with urban disaster management systems before AVs are released into “the wild” would reduce stakeholder resistance and the technical challenges associated with such laws. The longer we as a society wait for such standards, the more costly and more difficult they will be to implement.

### 1.1. Private Vehicle Use Following Earthquakes

Major earthquakes often result in trauma, injuries, and death [4]. A salient example was the 2008 Wenchuan earthquake in Sichuan, China that caused more than 370,000 casualties, leaving many people buried in the ruins of buildings. After that disaster, the Chinese government made major investments in both protocols and specialized vehicles to augment future rescue efforts. In the Sichuan province, civilian vehicles proved to be a major obstacle for rescue efforts. At the time, private ownership of cars was increasing rapidly, and they clogged the roads while also complicating rescue efforts as automobile owners attempted to rescue loved ones and belongings. Officials did not fully anticipate the negative effect that automobiles would have on hindering access of emergency vehicles to victims. With this disaster in the rearview mirror, it was possible to design roadways onto which emergency vehicles could be better integrated.

### 1.2. The Current State of Development of AVs

Today, many commercially-available vehicles offer features that allow remote access to built-in semi-autonomous systems (e.g., Tesla’s Autopilot). These systems, while only partially autonomous, could be used to remotely navigate the vehicle were the manufacturer to permit the government to do so. Presently, fully autonomous vehicles are undergoing testing on the roads in cities in China, Germany, Japan, Sweden, the UK and the US for commercial use [5], and commercial autonomous trucks are allowed to operate on highways. In the Sichuan Province of China autonomous aerial vehicles have been developed for use in an earthquake scenario for the purposes of rebuilding damaged communication networks.

Were another earthquake to occur, it would theoretically be possible to use existing semi-autonomous systems to clear roadways to improve emergency vehicle access. As more advanced autonomous features are added to privately owned vehicles, privately-owned vehicles can become an asset in a disaster situation, rather than a liability.

While it is unclear whether currently available semi-autonomous vehicles can be usefully deployed for disaster management, it is important to consider the use case now so that effective policies can be developed as self-driving technology advances.

### 1.3. Our Hyptothetical Case Study

After an earthquake, there are often injured victims who are trapped under debris and awaiting rescue. The time required to locate, evacuate, and stabilize victims is the primary determinant of the total number of casualties [6]. In this simulation study, we consider the use of AV systems for two basic purposes: (1) transporting emergency rescue personnel to disaster areas; and (2) relocating the victims to medical care facilities.

This paper therefore poses a relatively simple thought experiment examining the cost-effectiveness of a policy directed at remote, government control of AVs during a major earthquake. The purpose of this paper is not to address the political implications associated with government control of private vehicles, but rather to examine the larger potential societal value of broader use case scenarios as governments develop AV technology. However, we discuss the viability of our hypothetical scenario in different cultural contexts in the discussion.

Our model considers a scenario in which the costs are maximized but the benefits are minimized to ensure that any possible benefits measured are conservative. Under these constraints, if regulating AVs in disaster management is found to be cost-effective, then we can be reasonably confident that legislation that integrates AVs into disaster management plans would be associated with favorable returns on the investment.

## 2. Methods

### 2.1. Overview and Definitions

We built a Markov model comparing an intervention arm that used government control of AVs during an earthquake as part of a disaster response plan and a status quo arm in which the vehicle owner maintained control. Many urban areas worldwide (e.g., major Chinese cities, New York City, and Rio de Janeiro) have disaster management offices in which operations are centrally coordinated, and video feeds are obtained from both local cameras and satellite images. Our model assumes a relatively advanced center in which officials have real-time data on road conditions, collapsed buildings, and damaged infrastructure. We also include estimates of a range of costs including regulatory, legislative, and added technology costs.

Our model uses the US as a case scenario as a framework for standardizing costs. The first few hours following the earthquake are critical for saving lives [7,8], and we assumed that the survival rates of the two arms would converge after this time period. From a societal perspective, we included the costs of using AVs, regulatory fees, medical costs, as well as productivity loss due to early death. The quality-adjusted life year (QALY) was used as a health outcome measure. One QALY is roughly equal to one year of life spent in perfect health. To calculate the incremental cost-effectiveness ratio (ICER), we divided the additional costs associated with AVs by the QALYs gained. A 3% discount rate was used based on recommendations of the Second Panel on Cost-effectiveness in Health and Medicine [9]. We ran the model in TreeAge Pro 2013 (TreeAge software, Williamstown, MA, USA).

### 2.2. Patient and Public Involvement

There is no patient involved in the study. All the data concerning health outcome was from the literature.

### 2.3. Decision Analysis Model

**Figure 1:**
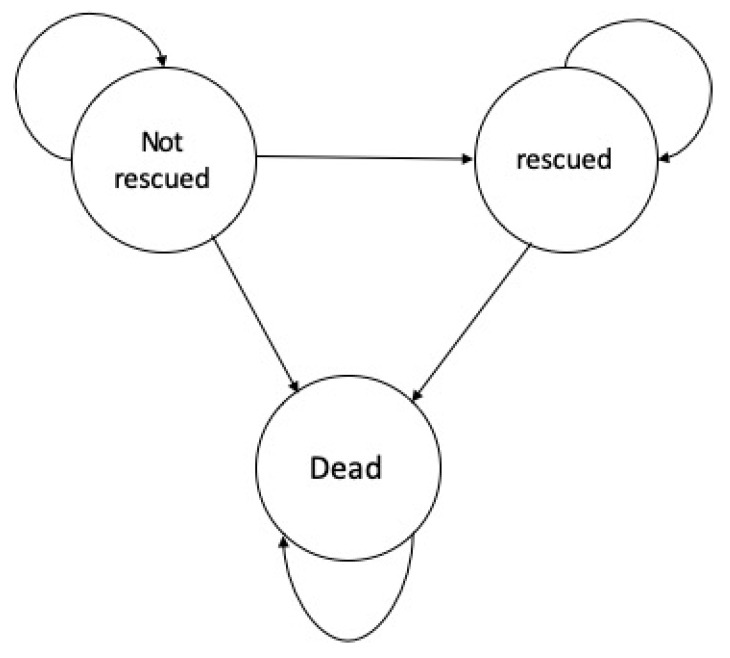
Markov model diagram of autonomous vehicles used in earthquake emergency rescue.

A Markov model describes how a hypothetical cohort transitions between different health states across model cycles. It is useful and suitable for the earthquake emergency rescue scenario, as the trapped victims may transition from the state of “not rescued” to “rescued” or “death” over time. In this research, the Markov model incorporated three health statuses: trapped and alive (“not rescued”), rescued and alive (“rescued”), and dead (Figure 1). All the simulated victims started from “not rescued”. They had a chance of being “rescued” or “dead”. The simulated participant in the intervention arm had a higher rescue rate than in the status quo arm. In addition, we assumed that people who were rescued were more likely to survive than the ones who remained trapped. The cycle length of our Markov model was 6 h.

### 2.4. Costs

Monetary costs were adjusted to constant 2017 US dollars and are listed in Table 1. Medical costs and productivity loss due to earthquake-related injury and fatality were obtained from Kleinhenz and Associates [10]. The costs of utilizing AVs include fixed and variable costs. As AVs are already available for use in the US, we did not incorporate their costs into our analyses. Other fixed costs such as the regulatory fee, wireless communication, and information system were modeled in our analyses. The variable costs included the maintenance, repair, and fuel costs. All the cost data were obtained from the Earth Institute at the Columbia University [11]. The regulatory costs of AVs were provided by the Competitive Enterprise Institute [12]. This organization estimates regulatory costs very broadly, and probably represents a high-end estimate of the total cost of any government regulation. More details are available in their report. The regulatory costs per simulation unit of our model was calculated by dividing the total costs by the number of the earthquake-affected persons.

Variable costs for AV include $0.525/mile, and were calculated based on distance between the disaster area and the nearest hospitals. The distance data were derived from the National Center for Health Statistics Research Data Center [13]. As it is likely the roads would be heavily damaged by the earthquake, we assumed that vehicle repair costs would need to be included in addition to operation costs. Conservatively, for the status quo arm we assumed that there were no vehicle-related costs.

### 2.5. Health Utility

The earthquake victims, especially those who were trapped, may suffer multiple types of trauma such as fractures, acute renal failure, and crush syndrome. According to the literature, it is assumed that the earthquake survivors’ quality of life score is 0.68 [14]. The formula below was used to calculate the earthquake survivors’ QALY.

$$\mathrm{QALY}\;=\;\sum_{n=1}^{k}U/{\left(1+r\right)}^{n}$$

where *k* represents the average remaining life time for the survivors. The median age of our hypothetical cohort was 37.8 years old and was subtracted from the life expectancy in the United States, or 78.9 years. *U* represents the health utility which is 0.68 in this scenario. r represents the discount rate.

### 2.6. Intervention Effect

To date, there is no available data capturing on-time performance of AVs in a disaster scenario. However, humans must verbally communicate and are prone to error. More importantly, untrained “Good Samaritans” often enter disaster situations with little coordination, and can slow emergency management operations down. Therefore, for this model we took a highly conservative approach, and assumed the same 5% on-time performance differential as in normal operating circumstances [15]. Since the survival rate of earthquake victims increases with faster rescue times [6], the AVs would save additional lives by reducing delays in rescue time.

Additionally, it is worth noting that no driver is needed to operate AVs, opening more space and resources for transporting injured victims. AVs could potentially increase the carrying capacity and allow rescue workers to focus efforts on stabilization rather than driving. Moreover, one of the key benefits of using private AVs in rescue operations is increasing the total number of emergency vehicles available. To explore the real capability of AVs used in disaster emergency rescue, 10% and 15% improvement of rescue efficiency were also modelled in this research as a sensitive analysis.

### 2.7. Probabilities

Table 2 shows both the rescue rates and survival rates used in the model. The time dependent rescue rate was generated based on a mathematical model developed by Oliveira et al. [16]. In their model, the rescue rate is calculated as a function of total number of casualties trapped against the amount of time passed since the event. For the purposes of this model, we assumed that the total number of casualties trapped was 1000 based on existing literature [17]. This serves as a starting point for estimating the scale of earthquake associated costs without actually impacting the ICER. That is, if a disaster is a degree larger or smaller, the ICER remains the same, but the total costs and lives saved differ according to the geographic context. From the literature, we assume 120 h as the status quo arm, while the intervention arms included 114 h as a 5% improvement, 108 h for 10%, and 102 h for 15% improvement (Table 2). Furthermore, we apply the survival rate into the rescue rate function for victims who may die during the rescue process. As a result, for the status quo and 5% improvement situation, there are no trapped victims awaiting rescue after 24 h, meaning there are four cycles in the model. Similarly, there are 18 h or three cycles in the 10% and 15% improvement situation model.

As a result, for both arms, we modeled four cycles (for a total of 24 h). The survival rate of both rescued and trapped victims is based on data collected from a report published by Keishi Shiono, which shows victim survival rates over time [6].

### 2.8. Sensitivity Analysis

We conducted a series of one-way sensitivity analyses along with the Monte Carlo simulation to test the reliability of our model. In the Monte Carlo simulation, a triangular distribution and Gamma distribution were used for variables. The random error associated with an estimate for the values were included within a plausible range.

## 3. Results

The cost of deploying AVs for earthquake response was $90,139 relative to $87,869 in the status quo arm (Table 3). Therefore, the use of AVs produced an incremental cost of $2269 (95% credible interval (CI) = $-12,985–$8959). Victims lived 7.33 QALYs in the intervention arm compared to 7.20 QALYs in the status quo arm, resulting in an incremental QALY gain of 0.13 (95% CI = −0.73–2.19) when the government is allowed to control an AV. The ICER was $16,960 per QALY gained (95% CI = cost-savings $69,065 per QALY gained).

As shown in Table 4, the sensitivity analysis suggests that the most influential variable in the model is rescued victims’ survival rate, followed by the discount rate, AV costs, productivity loss and buried victims’ survival rate. The rescued victims’ survival rate is the most sensitive variable in this model, for which a 25% increase in survival would decrease the ICER to $9609/QALY; whereas a 25% reduction would increase the ICER to $40,472/QALY. If we increase the discount rate, then the ICER changes from $9685/QALY to $22,960/QALY. If the AV costs change, the ICER also varies between $10,475/QALY to $23,446/QALY. Looking at the productivity loss and buried victim’s survival rate also indicates relative sensitivity to the values test (Table 4).

Furthermore, we also changed the 5% efficiency improvement of AVs to 10% and 15% as a sensitivity analysis. As shown in Table 3, the ICER is 2227 for 510% improvement and it is cost saving in the 15% improvement situation.

**Figure 2:**
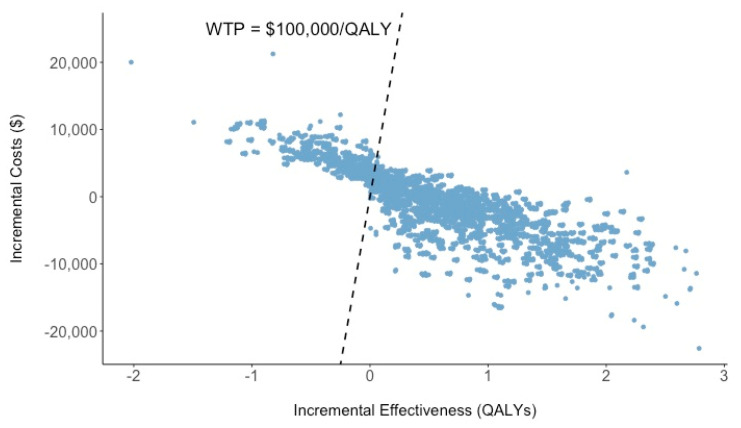
Cost-effectiveness plane for using automatous vehicles vs. human-driven vehicles. Abbreviations: QALY = quality-adjusted life year; WTP = willingness-to-pay.

The incremental cost-effectiveness plane is presented in Figure 2, in which each dot represents an incremental cost versus the incremental effectiveness associated with 10,000 Monte Carlo simulations within our comparative Markov model that evaluates deploying AVs versus the status quo. About 70%, of the simulations fall under a $100,000/QALY gained willingness-to-pay threshold.

## 4. Discussion

At present there are roughly 20 million semi-autonomous vehicles on the road [18,19], and AVs are already safer than HPVs under ideal road conditions [20]. Therefore, one might reasonably expect that they will be introduced “in the wild” within a decade in some areas. Autonomous vehicles offer tremendous potential for augmenting rescue efforts and allowing personnel and resources to be diverted towards stabilizing patients. Institutions like the Federal Emergency Management Agency (FEMA) have recognized the need to incorporate evolving technologies into disaster rescue management plans [21]. We find that it would be highly cost-effective to integrate AV control into existing urban disaster management command centers. Doing so is cheaper than treating hypertension or diabetes with medications, for example [20].

The results of our study are meant to be a starting point to galvanize the Congressional Budget Office (CBO) and FEMA to consider legislative efforts that ensure the proper use of AVs in disaster management.

### 4.1. Legal and Ethical Considerations

Regulations that allow the government to remotely operate privately-owned AVs could raise a number of legal and ethical concerns among private vehicle owners. The national responses to the COVID-19 crisis provide some context for understanding the resistance that such legislation might evoke. South Korea was able to adopt the widespread use of a technology that tracked individual’s test status and geographic location. This technology has been highly effective in utilizing private data for the public good, as it has allowed the government to effectively test and contact trace, rapidly quelling the epidemic. China has existing laws that allow individual privacy concerns to be set aside for the greater social good in the context of a national emergency. These laws would permit the deployment of AV technology for disaster management today. The US has several states that enact laws which will help to promote the wide use of autonomous vehicles. One could reasonably expect similar acceptance or resistance to privacy invasions to government control of AV technology.

Governments should be deliberate in building on the previous use of technologies in disaster responses. For example, the U.S. Army Corps of Engineers used remotely operated vehicles in the 2010 Haiti earthquake [22]. Our recommendation is that legislators consider working with the CBO and FEMA to devise concrete plans for the use of AV in future disaster management efforts.

### 4.2. Limitations

Our study made conservative assumptions regarding the life-saving ability of AVs and generous assumptions regarding the regulatory costs to ensure that probabilities were weighted against adoption of a rule in which governments are allowed to control private vehicles. Even so, it is likely that our oversimplified scenario overlooks some costs associated with AV legislation and infrastructure development. It is also important to note that there are other technologies, such as artificial intelligence, that are currently being integrated in to disaster management systems.

Given the constraints of estimating benefits and inflated costs, precision is a limitation of this study. In light of that, our results indicate that there is huge potential for government control of AVs to save money and lives in the event of an earthquake.

## 5. Conclusions

This research explores the costs and benefits associated with legislation that allows the government to take control of private AVs following an earthquake. Our paper simply presents objective estimates of the costs relative to the quality-adjusted life years gained were any such legislation to be introduced. The result shows AVs controlled by the government can be widely used in disaster emergency rescue action including disaster management which may greatly improve the efficiency of the recue action.

## Figures and Tables

**Table 1:** Annual Costs per person used in the Markov model evaluating autonomous vehicles (AVs) use in the disaster rescue and evacuation.

Variable	Base	High	Low
AV gas ^a^, $/mile	0.16	0.2	0.12
AV maintenance and repair ^a^, $/mile	0.05	0.06	0.04
Overhead costs ^a,b^, $	1066.18	1332.73	799.64
AV costs/person, $	3471	4339	2603
Medical cost ^c^, $	1001.14	1251.43	750.86
Value of lost life ^c^, $	176,482.98	220,603.73	132,362.24
Regulation fee ^d^, $	3204.58	4005.73	2403.44

a: the AVs cost per person was gained from Jordan, William C. 2012 [11] Cost/person = (cost/vehicle·mile) × D/N. D is the average distance form disaster area to nearest hospitals which is 2.5 miles from literature. N is the number of seats in a vehicle and we assume there are four seats in an AV. b: overhead costs contain wireless communication, information system and other costs. c: data is collected from Kleinhenz and Associates. 2014 [10]. d: data is collected from Clyde Wayne Crews Jr. 2016 [12].

**Table 2:** Rescue rates and survival rates in the Markov model for autonomous vehicles vs. human-driven vehicles using in earthquake rescue.

Time (h)	Rescued Victims’ Survival Rate (%)	Trapped Victims’ Survival Rate (%)	Rescue Rate with Man-Drive Vehicles (%)	Rescue Rate with Automated Vehicles of 5% Improvement (%)	Rescue Rate with Automated Vehicles of 10% Improvement (%)	Rescue Rate with Automated Vehicles of 15% Improvement (%)
0	100	100	0	0	0	0
6	96	60	42	44	45	47
12	90	44	56	58	59	62
18	84	38	94	99	100	100
24	78	32	100	100	0	0

**Table 3:** Costs, life years gained and incremental cost effectiveness ratio of using vs. not using autonomous vehicles.

Conditions	Costs ($)	Incremental Costs ($)	QALY	Incremental QALY	ICER ($/QALY)
Not using AV in earthquake rescue	87,869	-	7.2	-	-
Using AV in earthquake rescue (5% improvement)	90,139	2269	7.33	0.13	16,960
Using AV in earthquake rescue (10% improvement)	88,619	750	7.54	0.34	2227
Using AV in earthquake rescue (15% improvement)	86,976	−893	7.76	0.56	Cost saving

Abbreviations: AV = autonomous vehicle; ICER = incremental cost effectiveness ratio; QALY = quality-adjusted life year.

**Table 4:** Result of one-way sensitivity analysis for Markov model of using vs. not using autonomous vehicles for post-earthquake rescue.

Variable	ICER ($/QALY)	
Low: −25%; High: +25%	High	Low
Rescued victims’ survival rate	9609	40,472
Discount rate	22,960	9685
AV costs	23,446	10,475
Productivity loss	14,694	19,227
buried victims’ survival rate	20,927	17,921

Abbreviations: AV = autonomous vehicle; ICER = incremental cost effectiveness ratio; QALY = quality-adjusted life year.

## Data Sharing Statement

All data relevant to the study are included in the article and collected from the literature. All data can be shared in public.

## References

[1] Energy & Commerce Committee The SELFDRIVE Act. https://energycommerce.house.gov/selfdrive/.

[2] U.S. Department of Transportation Federal Automated Vehicles Policy. https://www.transportation.gov/AV.

[3] State of California Department of Motor Vehicles Deployment of Autonomous Vehicles for Public Operation. https://www.dmv.ca.gov/portal/dmv/detail/vr/autonomous/auto.

[4] Zhang L., Liu X., Li Y., Liu Y., Liu Z., Lin J., Shen J., Tang X., Zhang Y., Liang W. (2012). Emergency medical rescue efforts after a major earthquake: Lessons from the 2008 Wenchuan earthquake. Lancet.

[5] Lerman E.B., Suhareva S.V., Teslova S.A. (2020). Socio-economic aspects of the introduction of self-driving vehicles in an urban environment. IOP Conf. Ser. Mater. Ence Eng..

[6] Shiono K., Krimgold F. (1989). A computer model for the recovery of trapped people in a collapsed building: Development of a theoretical framework and direction for future data collection. Proceedings of the International Workshop on Earthquake Injury Epidemiology for Mitigation and Response.

[7] Feng C.-M., Wang T.-C. (2003). Highway emergency rehabilitation scheduling in post-earthquake 72 h. J. 5th East. Asia Soc. Transp. Stud..

[8] Wyen H., Lefering R., Maegele M., Brockamp T., Wafaisade A., Wutzler S., Walcher F., Marzi I. (2013). The golden hour of shock–how time is running out: Prehospital time intervals in Germany—A multivariate analysis of 15, 103 patients from the TraumaRegister DGU^®^. Emerg. Med. J..

[9] Sanders G.D., Neumann P.J., Basu A., Brock D.W., Feeny D., Krahn M., Kuntz K.M., Meltzer D.O., Owens D.K., Prosser L.A. (2016). Recommendations for Conduct, Methodological Practices, and Reporting of Cost-effectiveness Analyses: Second Panel on Cost-Effectiveness in Health and Medicine. JAMA.

[10] Kleinhenz & Associates Economic Benefit Cost Analysis: CoreFirst vs. Standard Retrofit..

[11] Jordan W.C. (2012). Transforming Personal Mobility. http://wordpress.ei.columbia.edu/mobility/files/2012/12/Transforming-Personal-Mobility-Aug-10-2012.pdf.

[12] Crews C.W. (2017). Ten Thousand Commandments An. Annual Snapshot of the Federal Regulatory State.

[13] National Center for Health Statistics Research Data Center “Distance to Nearest Hospital” Files. https://www.cdc.gov/nchs/ahcd/previous_new_ahcd.htm.

[14] Khachadourian V., Armenian H.K., Demirchyan A., Goenjian A. (2015). Loss and psychosocial factors as determinants of quality of life in a cohort of earthquake survivors. Health Qual. Life Outcomes.

[15] Hickman M. (2004). Bus Automatic Vehicle Location (AVL) Systems.

[16] Oliveira C.S., Roca A., Goula X. (2007). Assessing and Managing Earthquake Risk: Geo-scientific and Engineering Knowledge for Earthquake Risk Mitigation: Developments, Tools, Techniques.

[17] Oliveira C.S., Roca A., Goula X. (2006). Assessing and Managing Earthquake Risk.

[18] GovTrack (2017). H.R. 3388: SELF Drive Act. https://www.govtrack.us/congress/bills/115/hr3388/summary.

[19] Smith A., Anderson M. American’s Attitudes Towards Driverless Vehicles. http://www.pewinternet.org/2017/10/04/americans-attitudes-toward-driverless-vehicles/.

[20] Freedman I.G., Kim E., Muennig P.A. (2018). Autonomous vehicles are cost-effective when used as taxis. Injury Epidemiol..

[21] The U.S. Federal Emergency Management Agency (FEMA) (2018). A Proposed Research Agenda for the Emergency Management Higher Education Community.

[22] Guizzo E. (2011). Japan Earthquake: Robots Help Search for Survivors. https://spectrum.ieee.org/automaton/robotics/industrial-robots/japan-earthquake-robots-help-search-for-survivors.

